# A Worm-like Soft Robot Based on Adhesion-Controlled Electrohydraulic Actuators

**DOI:** 10.3390/biomimetics9120776

**Published:** 2024-12-20

**Authors:** Yangzhuo Wu, Zhe Sun, Yu Xiang, Jieliang Zhao

**Affiliations:** 1 School of Mechanical Engineering, Beijing Institute of Technology, Beijing 100081, China; wyz2022163@163.com (Y.W.); 3120240433@bit.edu.cn (Y.X.); 2 Beijing Institute of Astronautical Systems Engineering, Beijing 100076, China; sunzhe19881103@163.com

**Keywords:** worm-inspired, soft robot, electrohydraulic force, biomimetic wet adhesive

## Abstract

Worms are organisms characterized by simple structures, low energy consumption, and stable movement. Inspired by these characteristics, worm-like soft robots demonstrate exceptional adaptability to unstructured environments, attracting considerable interest in the field of biomimetic engineering. The primary challenge currently involves improving the motion performance of worm-like robots from the perspectives of actuation and anchoring. In this study, a single segment worm-like soft robot driven by electrohydraulic actuators is proposed. The robot consists of a soft actuation module and two symmetrical anchoring modules. The actuation modules enable multi-degree-of-freedom motion of the robot using symmetric dual-electrode electrohydraulic actuators, while the anchoring modules provide active friction control through bistable electrohydraulic actuators. A hierarchical microstructure design is used for the biomimetic adhesive surface, enabling rapid, reversible, and stable attachment to and detachment from different surfaces, thereby improving the robot’s surface anchoring performance. Experimental results show that the designed robot can perform peristaltic and bending motions similar to a worm. It achieves rapid bidirectional propulsion on both dry and wet surfaces, with a maximum speed of 10.36 mm/s (over 6 velocity/length ratio (min^−1^)).

## 1. Introduction

The need to perform detection tasks under size constraints or in extreme environments, such as military reconnaissance, post-disaster search and rescue, pipeline inspection, and endoscopic procedures, has driven interest in developing robots that can adapt to a variety of tasks, particularly in repetitive conditions. Although various mobile robots have been proposed with different modes of motion—such as wheel type [1,2], leg type [3,4], track type [5,6], and snake type [7,8]—they face challenges in achieving rapid propulsion and effective interaction with environmental interfaces in environments such as narrow gaps and openings, due to the use of motors and rigid drive components. An ideal detection device must exhibit high adaptability to complex environments, as well as stable and efficient anchoring capabilities. With the development of new materials, manufacturing techniques, and actuation technologies, soft robots—known for their high compliance and ability to interact effectively with unstructured environments—present an increasingly promising solution [9].

Taking advantage of the high flexibility and compliance of soft robots, current research in soft robotics focuses on improving the adaptability of robots in complex, dynamic environments, moving toward integrated designs that more closely mimic biological organisms in nature. In recent years, extensive research has focused on mimicking the multifunctionality of natural organisms by simulating biological motion patterns such as peristalsis [10,11], bending [12,13], and twisting [14]. Earthworms and caterpillars are commonly used as model organisms for soft robots. The peristaltic gait observed in these organisms is considered to be one of the most effective locomotion strategies for navigating unstructured terrain [15,16,17]. Earthworms, for example, can move quickly through narrow cracks or soil. Composed of multiple identical segments, they maintain structure through a fluid-filled coelom that functions as a hydrostatic skeleton. Earthworms achieve peristaltic locomotion by alternating segment contraction and extension, facilitated by muscle contraction and variable friction generated by their setae [18,19].

The stable and efficient locomotion of earthworms has inspired the design of numerous worm-like soft robots for applications such as soil exploration and endoscopic inspections. The key challenges in developing these robots are the designs of their drive technology and anchoring mechanisms. A variety of worm-like soft robots have been proposed, driven by different actuation technologies, including those using pneumatic actuators, shape memory alloys (SMAs), dielectric elastomers (DEs), and ionomeric polymer-metal composites (IPMCs). Pneumatic actuation is a well-established drive technology for worm-like robots [20,21,22,23]. While these robots offer good flexibility and controllability, their portability is hampered by the limitations of pneumatic actuators and the need for additional components such as air pumps and valves. Fluid flow through the pipelines leads to efficiency losses and limits the robot’s range of motion. In order to adapt to narrow spaces, researchers have developed a series of compact worm-like robots that utilizing the extensibility of SMAs [24]. Compared to other actuation methods, worm-like robots require a cooling period for the natural cooling of SMAs, which limits the continuous motion performance of SMA-driven robots [24,25]. DEAs offer advantages such as high electromechanical efficiency, fast response times, and precise control. However, dielectric elastomer materials are susceptible to failure due to dielectric breakdown, which limits the service life of a DEA robot. Moreover, increasing the driving voltage frequency may damage the connection between the DE and the rigid frame, thereby limiting the robot’s speed [26,27]. Furthermore, the robotic prototypes in these studies lack the capability to adjust their direction. IPMCs offer advantages such as low driving voltage and large deformation [28], but their slow response time limits continuous motion performance. In addition, repeated bending causes cracks in the electrode materials, reducing the robot’s service life.

In addition to the challenges of actuation technology, achieving fast and reliable adhesion switching is a critical aspect of worm-like robots. Pipeline robots often use the expansion adhesion principle to attach to pipe walls [20,22,23,26]. However, this method is not suitable for ground crawling robots. In addition, soft structures inspired by octopus suckers, which rely on negative pressure adhesion, are prone to damage, compromising stable attachment [29,30]. Many animals in nature, such as geckos and spiders, exhibit exceptional adhesion abilities. They achieve dry adhesion using the van der Waals forces between the tips of their setae and the surface [31,32]. This has inspired the development of various biomimetic grippers and crawling robots [33,34,35]. These robots can achieve stable attachment on dry surfaces. However, existing biomimetic adhesive materials require substantial preloading to achieve high adhesion forces [36]. Furthermore, biomimetic surfaces based on setae structures have difficulty maintaining stable adhesion on moist substrates. Animals such as tree frogs and octopuses can maintain a stable attachment in moist or even liquid environments, a process known as wet adhesion. Inspired by this, researchers have developed various biomimetic wet adhesive surfaces [37,38]. However, these surfaces still face challenges, including low adhesion strength and slow switching times.

Recent innovations in electrohydraulic actuation technologies make it possible to overcome the performance limitations of traditional actuators [39,40,41]. To overcome these challenges, the development of multifunctional, high performance worm-like soft robots requires lightweight actuators with high output force, large strain, and fast response. Hydraulically amplified self-healing electrostatic (HASEL) actuators combine the advantages of fluid actuators and dielectric elastomer actuators (DEAs). They operate on the basis of electrostatic forces and fluid pressure, achieving effective deformation through localized fluid pumping. Compared to fluid actuators, HASEL actuators do not require an external source of compressed fluid or a pump. This reduces the fluid travel distance and consequently the response time. They also have superior self-healing capabilities compared to dielectric elastomer (DEA) actuators. And because they are voltage controlled, they offer excellent control accuracy.

Previous studies have successfully applied electrohydraulic actuators to inchworm-inspired crawling robots [42]. These actuators offer advantages such as low weight and high flexibility, opening up new possibilities for the design of soft crawling robots. Currently, inchworm-inspired crawling robots require additional parallel modules for directional control, which limits their ability to adjust the direction of propulsion. At the same time, the robot relies solely on the return force generated by the fluid flow to switch between motion states, which limits its frequency of operation. In addition, frictional anisotropy is achieved by using friction pads with different coefficients of friction, but maintaining effective friction on wet surfaces is a challenge. Active control of motion through friction can improve a robot’s anchoring stability and enable bidirectional motion, increasing its potential for autonomous mobility. In particular, applying a load to the robot increases its tendency to slip backward, resulting in a loss of stride of motion. As a result, enabling crawling robots to navigate more complex environments remains a challenge. To fully exploit the advantages of peristaltic organisms in nature, it is essential to develop multifunctional crawling soft robots with enhanced locomotion performance.

This paper presents a worm-like soft robot actuated by electrohydraulic actuators. The proposed soft robot is driven by an electrohydraulic actuator and features a biomimetic wet adhesive surface design based on hierarchical polygonal microstructures, enabling multi-degree freedom of motion and active control of the adhesion force. By rapidly moving a robot bidirectionally on a flat surface, we demonstrate its high adaptability to both dry and wet surfaces, demonstrating the robot’s performance and efficiency. The advances in propulsion performance of this novel soft crawling robot offer a wide range of potential applications, including military reconnaissance and environmental monitoring, and open up new possibilities for the further use of electrohydraulic actuators in soft robotics and biomimetics.

## 2. Materials and Methods

### 2.1. Biological Inspiration

**Figure 1:**
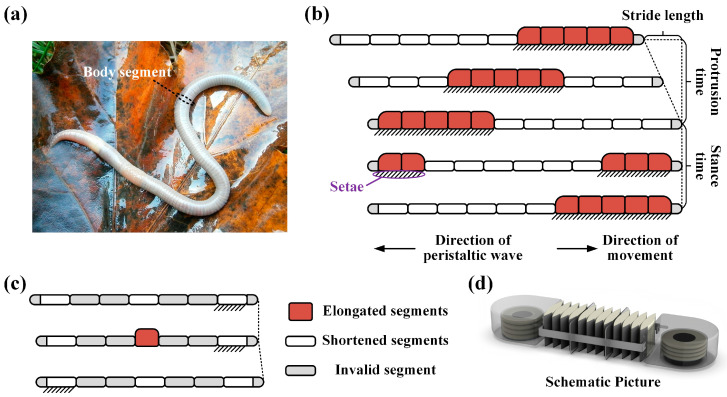
Physiological structure and locomotion mechanism of typical worms. (**a**) Earthworms are a typical worm organism. (**b**) A complete stride period of the peristaltic crawling. (**c**) Stride period of single-segment robot. (**d**) Concept of the designed worm-like soft robot.

This work is inspired by earthworms. Researchers have observed that earthworms are made up of several identical segments, each containing antagonistic circular and longitudinal muscles, as well as setae that can extend and retract. These antagonistic muscles allow the segments to switch between the extended and contracted states [19]. In addition, as the earthworm’s segments contract, the setae on its periphery protrude, enhancing its anchoring capability with the ground [43]. The periodic peristaltic movement of the earthworm consists of protrusion and stance time. During the protrusion time, the posterior segments anchor to the ground while the anterior segments move forward. During the stance time, the anterior segments anchor while the posterior segments retract. The contraction of each segment is sequentially transmitted posteriorly, creating a peristaltic wave that propagates in the opposite direction of movement (Figure 1b) [44]. As shown in Figure 1c, the complete motion cycle can be achieved by alternately actuating at least three segments. Single-segment worm-like robots have the advantages of a simple structure and simple control.

### 2.2. Design of the Worm-like Soft Robot

**Figure 2:**
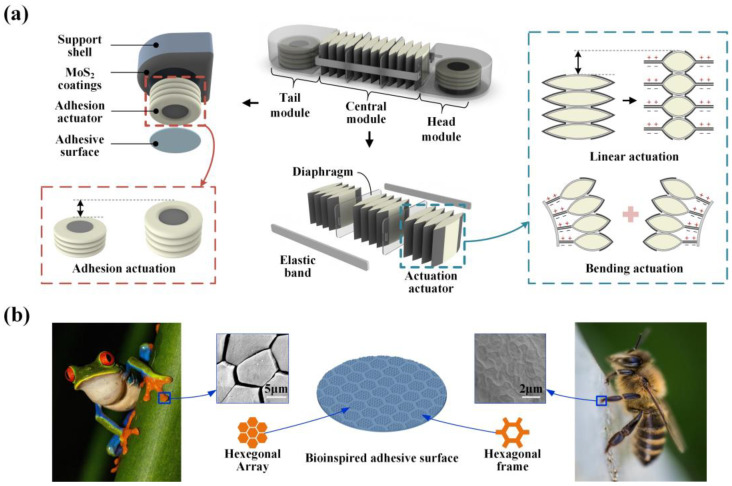
Design and structure of worm-like soft robot. (**a**) The structure of the robot consists of a central actuation module and two anchoring modules (tail/head module). (**b**) Hierarchical micro-nano adhesive surface inspired by honeybees and tree frogs.

To replicate the stable and efficient locomotion characteristics of worms, we propose a single-segment worm-like soft robot. By studying the worm’s physiological structure and locomotion mechanisms, we conclude that the key to effective biomimicry lies in simulating the deformation of the worm’s segmented muscles and its controllable anchoring function in relation to the environment. Studies show that earthworms have no skeletal structure; they maintain their shape through fluid-filled coelom cavities. This hydrostatic skeleton structure matches the local fluid flow characteristics of electrohydraulic actuators. Figure 2a illustrates the design concept of the robot, which consists of two fully symmetrical anchoring modules connected in series with a central actuation module. Most existing electrohydraulic actuators are designed to contract when actuated. However, for worm-like robots that require active propulsion, actuators need to extend during operation. A previously proposed inchworm-inspired robot uses the bending motion of the electrohydraulic actuator to achieve robot extension [42]. However, it requires a high activation voltage to initiate the system. To enable bidirectional movement and direction adjustment, the robot’s central module is formed by stacking several sets of rectangular electrohydraulic actuators with symmetrically distributed electrodes (Figure 2a). When an electrode on one side is activated, another side of the actuator pouch expands, causing the structure to bend. Activating electrodes on both sides results in linear strain (Figure 2a). Using this stacked, symmetrical, dual-drive electrode actuator, capable of both linear and bending strains, as the robot’s actuation actuator.

To improve the mobility of the worm-like robot, a stable and controllable anchoring mechanism is essential. While pipeline robots typically use an expansion adhesion principle to attach to the pipe wall, this method is not suitable for ground-based crawling robots. In addition, relying on materials with different coefficients of friction to create anisotropic friction offers limited reliability and limits the robot’s load capacity. The pull-in characteristic of the HASEL actuators provides a fast, bistable response [44], which meets the need for fast switching between ground contact states in adhesive actuators. Therefore, we designed an actively controllable adhesion module based on HASEL actuators, which consists of adhesion actuators, a support shell, and an adhesive surface. The adhesive actuator is made of a stack of circular HASEL actuators, with its top fixed to the inner side of the supporting shell, with an adhesive surface bonded to the bottom. In the initial state, the adhesive surface does not contact the ground. Activating the anchoring actuator induces a strain in the vertical direction, as shown in Figure 2a, causing the adhesive surface at the bottom to gradually contact the ground. The voltage is continuously increased until the supporting shell is fully lifted, reaching the maximum achievable preload (its own weight).

To enable adhesive surfaces to adapt to wet environments, we take inspiration from the attachment mechanisms of biological systems. Many organisms in nature have terminal adhesive pads with exceptional adhesive properties. Studies show that the toepads of tree frogs feature a hierarchical structure. The primary tier consists of polygonal pillars formed by epithelial cells, while the secondary tier is characterized by a hexagonal cavity arrangement. This structure not only enhances the adaptability of the frog’s toe pads to irregular, rough surfaces, but also facilitates the removal of excess fluid from the contact interface through the grooves around the pillars when fluid accumulation occurs. As a result, the frog-inspired biomimetic surface exhibits exceptional wet adhesion performance [45]. In addition, bees are organisms with remarkable attachment capabilities, capable of high-frequency attachment and detachment on surfaces such as moist interfaces, complex flower petals, and extremely fine plant stems. Liang et al. used SEM to examine the foot-end patterns of bees and discovered that the attachment surface features a polygonal patterned structure (Figure 2b). This cavity structure increases the shear adhesive force through the liquid self-sucking effects at the three-phase interface and the air-embolism effects [46]. We fabricated a biomimetic adhesive surface using polydimethylsiloxane (PDMS) with a tree frog-inspired primary structure and a bee-inspired secondary structure to enhance the adhesion of the HASEL actuator when it is in contact with the ground. We also applied a MoS_2_ coating to the underside of the support shell to reduce friction between the support shell and the ground when the actuator is not in contact (Figure 2b).

### 2.3. Analysis of Mathematical Models

**Figure 3:**
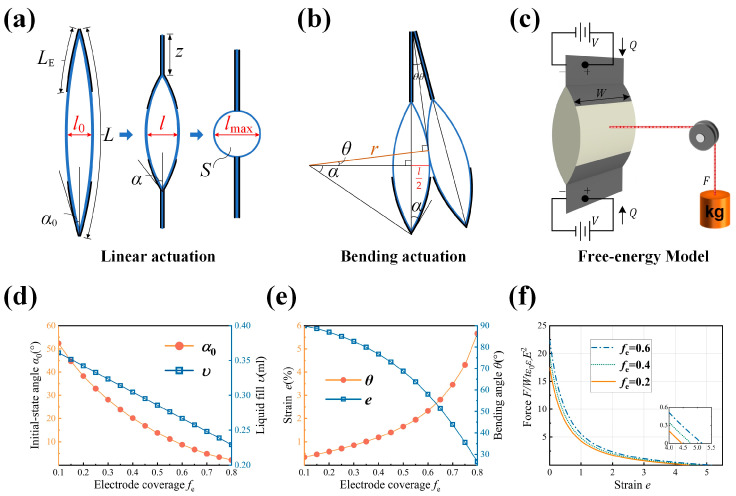
Analytical model of the actuation actuator unit. (**a**) Activation of electrodes on both sides resulting in linear motion. (**b**) Activation of electrodes on one side resulting in bending motion. (**c**) Free energy model of the actuator. (**d**) Initial-state angle and liquid fill amount of the actuation actuator for different electrode coverage. (**e**) Strain and bending angle of the actuation actuator unit for different electrode coverage. (**f**) Force-strain behavior of the actuation actuator unit for different electrode coverage.

For the actuation module, we focused on its continuous variation characteristics, which directly affect the motion pattern of the worm-like robot. For the anchoring module, we only utilized its bistable characteristics to control the switching of whether the adhesive surface is in contact with the ground. Therefore, in order to predict the response behavior of the actuation actuator and to guide its fabrication, this paper examines several factors that influence the quasi-static behavior of the actuator, with reference to the methodology described by Kellaris et al. [47]. The analysis models for the linear and bending strains of the actuation actuator are shown in Figure 3a,b. The cross-section of the actuator shell is of width *W*, length *L*, and the electrode coverage length on one side is *L*_E_. The bending stiffness of the film material is neglected. When a voltage is applied to the electrodes, the electrodes on both sides gradually zip together from the edges, with a contact length of *z*. As a result, the central angle of the actuator changes from 2*α*_0_ to 2*α*, inducing strain in the thickness direction of the actuator. Since the actuator is filled with an incompressible liquid of volume *υ*, the cross-sectional area *S* at the central of the actuator can be expressed as:

(1) $$S=L^{2}\frac{2\alpha_{0}-\sin\left(2\alpha_{0}\right)}{4\alpha_{0}^{2}}={\left(L-2z\right)}^{2}\frac{2\alpha -\sin\left(2\alpha \right)}{4\alpha^{2}}$$

The variation in actuator thickness is:

(2) $$l_{0}=L\frac{1-\cos\left(\alpha_{0}\right)}{\alpha_{0}}$$

(3) $$l=\left(L-2z\right)\frac{1-\cos\left(\alpha \right)}{\alpha }$$

Therefore, the linear strain *e* of the actuator is:

(4) $$e=\frac{l}{l_{0}}-1=\frac{L-2z}{L}\frac{\alpha_{0}}{\alpha }\frac{1-\cos\left(\alpha \right)}{1-\cos\left(\alpha_{0}\right)}-1$$

When the actuator’s cross-section becomes circular, further compression is not possible. The liquid volume corresponding to the point where the actuator’s cross-section exactly forms a circle upon electrode contact is defined as the actual filling volume. That is:

(5) $$S=\frac{{\left(L-2L_{E}\right)}^{2}}{\pi }$$

By combining Equations (1) and (5), the electrode coverage *f*_e_ is related to the initial angle *α*_0_ as follows:

(6) $$f_{e}=\frac{2L_{E}}{L}=1-\frac{\sqrt{\pi \left(2\alpha_{0}-\sin\left(2\alpha_{0}\right)\right)}}{2\alpha_{0}}$$

At this point, the actuator experiences its maximum strain:

(7) $$e_{\max}=\frac{l_{\max}}{l_{0}}-1=\sqrt{\frac{2\alpha_{0}-\sin\left(2\alpha_{0}\right)}{\pi }}\frac{1}{1-\cos\alpha_{0}}-1$$

With the actuator dimensions set to a length of *L* = 3 cm and a width of *W* = 2 cm, the relationship between electrode coverage and liquid fill is obtained as shown in Figure 3d.

When one side of the electrode is activated and fully adhered, the bending angle 2*θ* of two adjacent actuator units is also related to the electrode coverage. This relationship is derived from the geometric configuration shown in Figure 3b:

(8) $$r-\frac{r\cos\alpha }{\cos\theta }=\sin\theta \left(L_{E}+2r\sin\alpha -r\cos\theta \tan\theta -r\sin\alpha \right).$$

This results in the relationship between the linear strain and the bending angle between the two actuator units at different electrode coverage, as shown in Figure 3e. The maximum linear strain of the actuator unit increases as the electrode coverage increases, while the maximum bending angle decreases as the electrode coverage increases. In the actual fabrication process, higher electrode coverage is prioritized to achieve higher linear strain, as improving the robot’s propulsion speed is our primary focus. Improving the response speed of actuators, increasing actuator strain, and increasing the output force are three key strategies to optimize the locomotion performance of worm-like robots. To predict the output force of the actuator, a free energy model is established as shown in Figure 3c. The total free energy is considered to consist of both the mechanical and electrical energy stored in the actuator, together with the contribution from the external force *F* interacting with the actuator and the electrical energy exchanged with the constant pressure battery *V*. The electrodes on both sides are modeled as a parallel plate capacitor with capacitance *C*, giving an expression for the total free energy of the system.

(9) $$H=-QV+\frac{1}{2}CV^{2}+\left(l-l_{0}\right)F$$

At equilibrium, the free energy of the system is minimized with respect to *α*. By minimizing Equation (9), the state equation is solved:

(10) $$F=Wt\frac{\cos\left(\alpha \right)\left(\sin\left(\alpha \right)-\alpha \cos\left(\alpha \right)\right)}{2\sin\left(\alpha \right)\left(\alpha \;-\;\sin\left(\alpha \right)\right)}e_{r}e_{0}E^{2}$$

Here, *t* is the film thickness, *ε*_r_*ε*_0_ is the dielectric constant of the thin film material, and *E* is the electric field strength. Figure 3f shows the normalized stress–strain curve of the actuator at different electrode coverage. Changing the electrode coverage changes the y- and x-intercepts of the curves, which correspond to the maximum force generated by the actuator in the absence of displacement and the actuation strain when the actuator is unloaded, both of which increase with increasing electrode coverage. In the actual selection of design parameters, although the actuator has a higher theoretical strain when the electrode coverage rate exceeds 0.6, the theoretical volume of fluid inside the actuator decreases rapidly as the coverage rate increases. To achieve a given strain, a significant increase in the number of stacked actuators is required, which reduces the efficiency of actuator fabrication. Based on the above analysis, we selected the following actuator parameters for subsequent experiments: *L* = 3 cm, *W* = 2 cm, *f*_e_ = 0.6, and liquid fill amount *v* = 0.5 mL (Considering the need to expel the bubbles generated during the injection of the liquid, a value higher than the theoretical liquid fill is used).

### 2.4. Fabrication of the Worm-like Soft Robot

**Figure 4:**
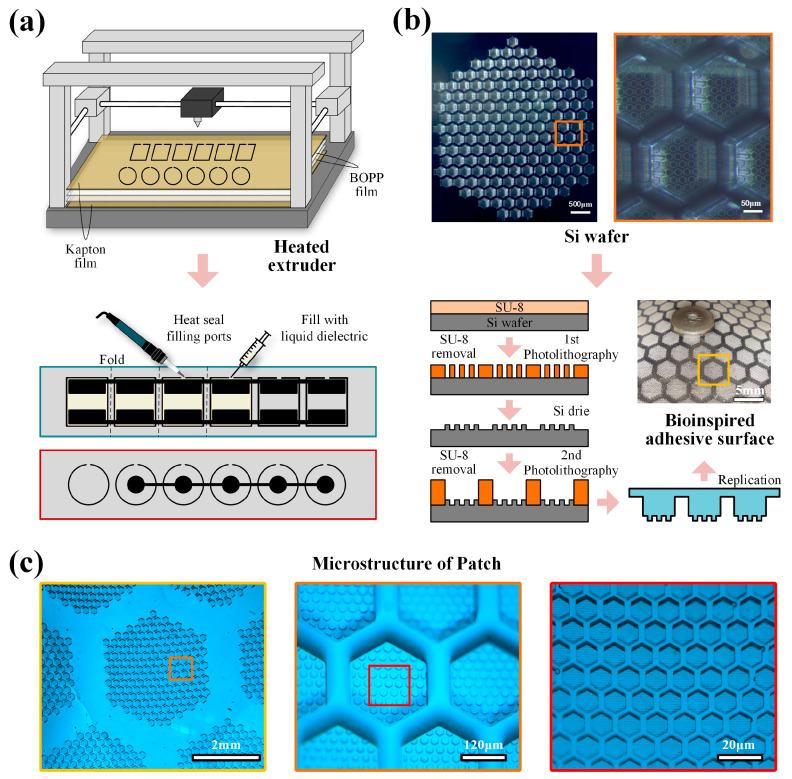
Fabrication of actuators and adhesive surface. (**a**) Fabrication process of the actuators. (**b**) Fabrication process of the biomimetic adhesive surface. (**c**) Microstructure of the biomimetic adhesive surface.

The fabrication of the electrohydraulic actuators follows the approach proposed by Mitchell et al. [41]. A CNC heat-sealing machine is used to seal two layers of BOPP film, creating a series of small bags with a filling port. Carbon-based graphene electrodes are screen printed on both sides of the bags. After each bag is filled with a specified amount of liquid dielectric, the filling port is sealed using an electric soldering iron. The use of this flexible, non-stretchable film eliminates the need for the fiber stiffness layers commonly required in pneumatic actuators, thereby simplifying the fabrication process. Folding the rectangular and circular bags results in a set of linear/bending actuators and adhesive actuators, as shown in Figure 4a. In order to achieve more uniform contact of the adhesive material with the surface, the lower adhesive actuator is not printed with electrodes. Folding the rectangular and circular pouches results in a set of linear/bending actuators and adhesive actuators, as shown in Figure 4a. To achieve more uniform contact of the adhesive surface with the ground, the bottom adhesive actuator is not printed with electrodes. The connection of the PET frames in the actuation actuators provides rigidity to the robot’s actuation module. In addition, a layer of silicone elastic tape is added to allow the actuators to quickly return to their initial state after activation.

The process of preparing the biomimetic wet adhesive surface, inspired by tree frogs and bees, is shown in Figure 4b. After the silicon wafer has been exposed, developed, and photoresist stripped, the silicon substrate is etched using deep reactive ion etching (DRIE) to produce a secondary hexagonal frame structure mold. The wafer is then subjected to a second round of photoresist spin coating, exposure, development, and photoresist stripping, resulting in a primary hexagonal prism structure mold. A PDMS precursor solution mixed at a 10:1 weight ratio is poured into a mold and heat cured to produce an adhesive surface. The sample is then examined under a microscope, revealing a well-defined primary hexagonal prism structure and a secondary hexagonal frame structure (Figure 4c).

## 3. Results

### 3.1. Performance of Actuation and Anchoring Module

**Figure 5:**
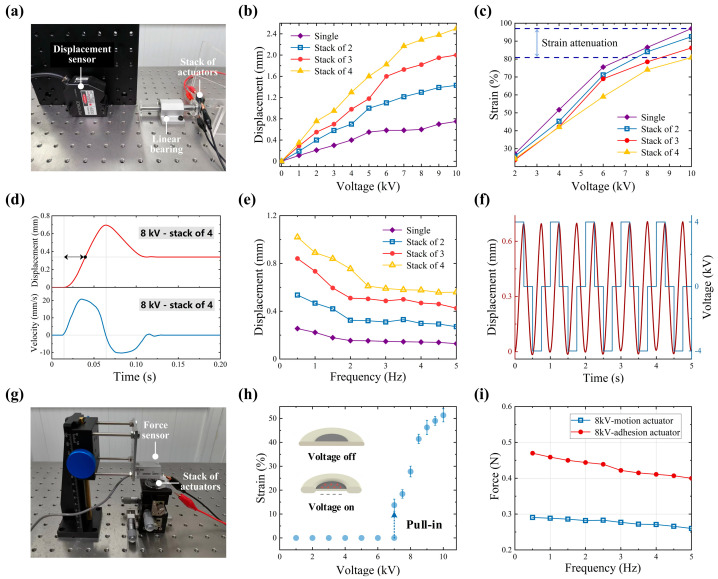
Response performance testing of the actuators. (**a**) Experimental setup for strain measurement. (**b**) Voltage–displacement curves. (**c**) Calculated voltage–strain curves. (**d**) Displacement and velocity of the Actuators. (**e**) Voltage–frequency curves. (**f**) Displacement response. (**g**) Experimental setup for force measurement. (**h**) Voltage–strain curves. (**i**) Frequency–force curves.

Based on the locomotion mechanism of the worm-like robot, it is clear that in actuation modules, improving the actuator’s response speed reduces the duration of one stride period in a worm robot and increasing the actuator’s strain increases the robot’s stride length. Both factors directly increase the robot’s propulsion speed. We began by testing the responsiveness of the actuation actuator. The displacement measurement setup is shown in Figure 5a, where the displacement of the actuators was constrained along the linear direction using linear bearings. A laser displacement sensor (Keyence, LK-H055, Shanghai, China) was used to measure the relationship between the linear displacement of actuators stacked in varying numbers and the applied voltage. The drive voltage was supplied by a high voltage amplifier (Aigtek, ATA-D61010, Shaanxi, China) capable of delivering a maximum output of 20 kVp-p. After measuring the initial thickness of the actuators with a micrometer, we calculated the relationship between their strain and the applied voltage, as shown in Figure 5a,b. The linear strain of the actuators decreased with increasing stack number, which is attributed to the uneven distribution of the actuator fluid. Nevertheless, all four sets of actuators showed an elongation of more than 80% at a voltage of 10 kV. Figure 5d shows the performance of the actuator during rapid expansion to its steady state position. We also investigated the dynamic response of the actuators under square wave voltage signals at different frequencies. To reduce the influence of electric charge residue on the response, the polarity of the square wave drive signal was reversed in each cycle. As shown in Figure 5e,f, the actuators exhibited stable dynamic responses.

In addition to focusing on the actuator’s strain, we also consider its output force. Although, when the adhesive actuators are not activated, only the 3D-printed support shell (which has a low friction coefficient) contacts the ground, sufficient output force is still necessary to drive the adjacent actuator groups as well as the head/tail modules at the non-anchored end. In the robot designed in this study, a set of stacked anchoring actuators and a supporting shell have a total mass of 20 g. The coefficient of friction for the ABS material is approximately 0.12. When a 100 g load is applied, the actuators’ theoretical output force must exceed 100 mN. Considering the stiffness of the electrohydraulic actuator shell and the need to overcome the elastic force of the components, the actual required output force exceeds the theoretical value. For the anchoring module, enabling the adhesive actuators to respond quickly to complete the switch between anchored states can minimize the robot’s pause time, allowing the next stride period to begin as soon as possible, thereby increasing the robot’s propulsion speed. To achieve the desired anchoring effect, the output force of the adhesive actuator should be maximized to support its own weight and the weight of the supporting shell (approximately 0.2 N in this study), thereby increasing the normal pressure on the adhesive surface. We measured the force output of the actuators using a force sensor (GSO-500, Transducer Techniques, Temecula, CA, USA), with the test setup shown in Figure 5g. As the adhesive actuators only start to move when the critical voltage is exceeded, we first tested their unique bistable pull-in characteristic, as shown in Figure 5h. Based on these test results, we determined the drive voltage. The force output of two sets of actuators was then measured under square wave voltage signals at 8 kV with varying frequencies, as shown in Figure 5i. The force output showed a negative correlation with the frequency of the voltage. These measurements also provide important reliability metrics for the subsequent anchoring modules. To prevent the structure of the robot’s head or tail shell from lifting and affecting the stability of its movement, the adhesive actuators inside the shell should be aligned approximately with the base of the shell when they reach their maximum stroke. Based on this requirement, the appropriate number of actuator stacks and the internal depths of the shell are determined. To achieve optimum anchoring performance, the output force of the adhesive actuators should be maximized to support both their own weight and the weight of the shell, thereby increasing the normal pressure.

### 3.2. Bioinspired Adhesive Surface Experiments

**Figure 6:**
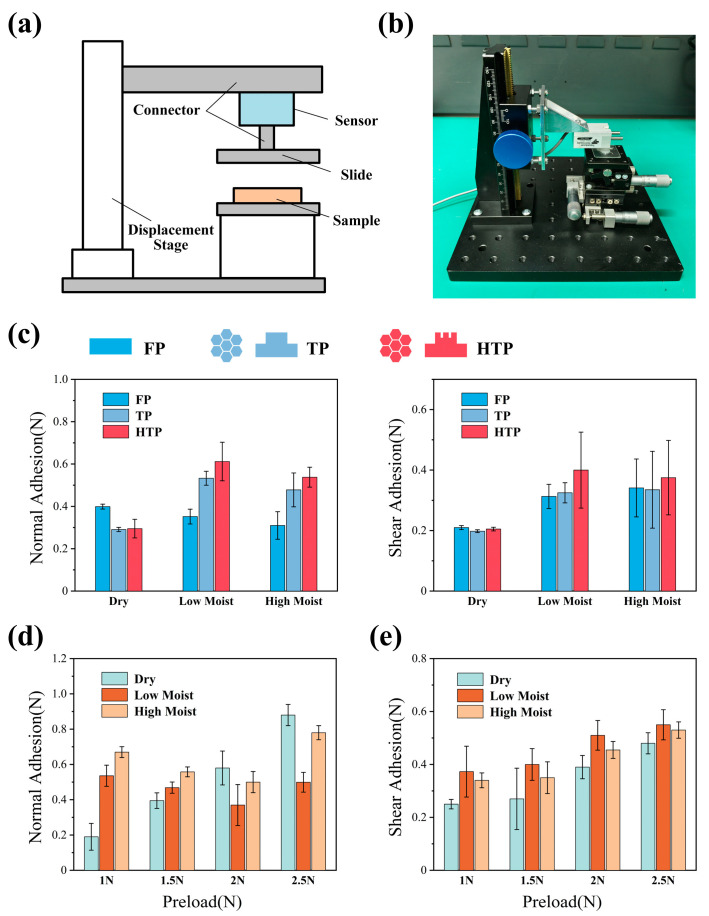
Bioinspired adhesive surface experiments. (**a**) Schematic of the configuration used to measure adhesion. (**b**) Adhesion measurement system. (**c**) Adhesion forces of FP, TP, and HTP in various surface conditions. (**d**,**e**) Preload–adhesion curves in various surface conditions.

The working mechanism of the anchor module dictates that the adhesive surface on the bottom of the actuator must provide strong shear adhesive forces to prevent slippage during the movement of the worm-like robot, while also allowing for rapid adhesion and release. We developed an adhesion force testing system to measure the shear and normal adhesive forces of the biomimetic surface under both dry and wet conditions. The setup essentially consists of a normal displacement stage, a force sensor, a test sample (10 mm × 10 mm), and a three-axis displacement stage (Figure 6b). The test procedure consists of three steps: (1) The test sample is first ultrasonically cleaned and dried, then mounted on the three-axis displacement stage. Different volumes of deionized water are applied to the center of the sample to simulate both dry and wet adhesion conditions. (2) The contact between the test substrate (glass slide) and the test sample is controlled by adjusting the displacement stage, which in turn controls the preload pressure. (3) The normal displacement stage is manually adjusted to release the sample until it completely separates from the substrate. This process is repeated, and the average value is calculated together with the error band. The test results shown in Figure 6c,d represent a comparison of three different patch types: a flat patch (FP), a single level hexagonal pillar structure inspired by tree frogs (TP), and a hierarchical structure inspired by both honeybees and tree frogs (HTP). Analyzing the results of the normal and shear adhesion force, it can be seen that under dry conditions, there is no significant difference in the adhesion force value. At this stage, the FP, with its larger contact area, shows superior adhesion performance. Under wet adhesion conditions, the HTP exhibits higher shear and normal adhesion force than both the FP and TP at low moisture levels. However, as the humidity increases, the adhesion force of the FP decreases sharply, while the HTP maintains a relatively high adhesion force. The adhesion force of the HTP consistently outperforms the TP under varying humidity conditions. The hierarchical structure, which incorporates a secondary hexagonal framework derived from the hexagonal pillar design inspired by the tree frog, significantly enhances the adhesive performance of the biomimetic adhesive surfaces. This improvement is attributed to the air-embolism effects created by the honeybee-inspired hexagonal cavities [46]. Unlike vacuum suction adhesion, these adhesive surfaces do not require a tightly sealed environment and have lower preload requirements.

### 3.3. Locomotion Mechanism and Control System

**Figure 7:**
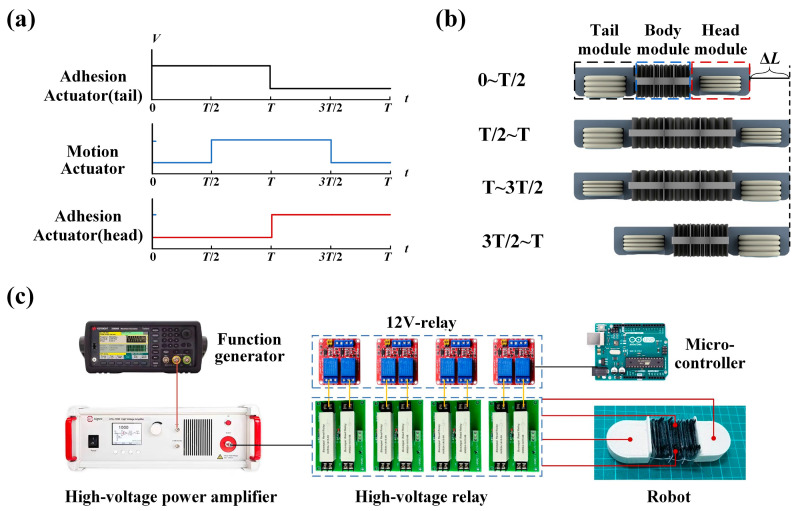
Design of the robotic actuation system. (**a**) Periodic control signals for the robot. (**b**) Locomotion principle of the worm-like robots. (**c**) The configuration of the control system.

The single segment worm-like robot operates on a relatively simple principle of motion. Each motion cycle of the robot consists of a series of state transitions through the following stages (Figure 7b).

(1) In the 0~T/2 phase, the tail adhesive actuators are activated, generating axial elongation that presses the adhesive surface against the ground for anchoring. The motion actuators and head adhesive actuators remain inactive.
(2) In the T/2~T phase, the tail and head adhesive actuators maintain their states, while the motion actuators are activated to extend or bend the robot’s head module forward.
(3) In the T~3T/2 phase, the working states of the tail and head adhesive actuators are switched, anchoring the head module to the ground.
(4) In the 3T/2~T phase, the tail and head adhesive actuators maintain their states, and the motion actuator’s charge is released, allowing it to return to its initial position under the restoring force of the elastic element, thereby pulling the robot’s tail module forward to elongate it.

Under ideal anchoring conditions, the robot avoids backward slippage and moves a certain distance in each cycle. By repeating the four steps, the robot can move forward continuously. Reversing the sequence of these steps results in backward movement.

### 3.4. Worm-like Robot Crawling Performance

To achieve the robot’s movement, a motion control system is established to activate four sets of actuators. The high voltage required to drive the actuators is supplied by a high-voltage amplifier (Aigtek, ATA-D61010, Shaanxi, China), which amplifies the voltage signal from the function generator. Each set of independently controlled flexible electrodes corresponds to a high-voltage relay (Boompai, HRM12-2A15, Shanghai, China), which controls both the voltage and polarity. For equipment safety, the on/off status of the high-voltage amplifier is managed by low-voltage relays. The power-up sequence of the multi-channel high-voltage drive circuits is controlled by signals from a microcontroller.

**Figure 8:**
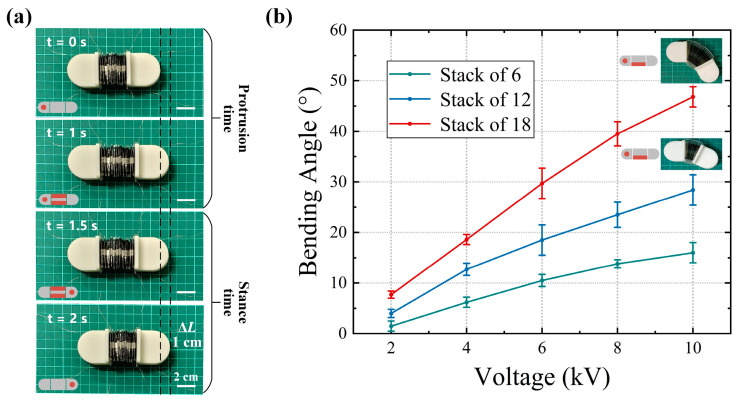
Basic motion of the worm-like robot. (**a**) Rectilinear locomotion over one stride period. (**b**) Bending locomotion of the robot.

Guided by the actuator module response results in Section 3.1, a high voltage of 8 kV was chosen to maximize the robot’s strain while avoiding dielectric breakdown. Figure 8a illustrates the robot’s linear motion during a complete stride period. The stable anchoring effect allows the robot to elongate up to 10 mm. Figure 8b shows the relationship between the bending angle and voltage (tail module anchored to the ground). When the actuator’s unilateral electrode is driven, the robot’s head, composed of 18 stacked actuator units, undergoes a significant deflection of 50°.

**Figure 9:**
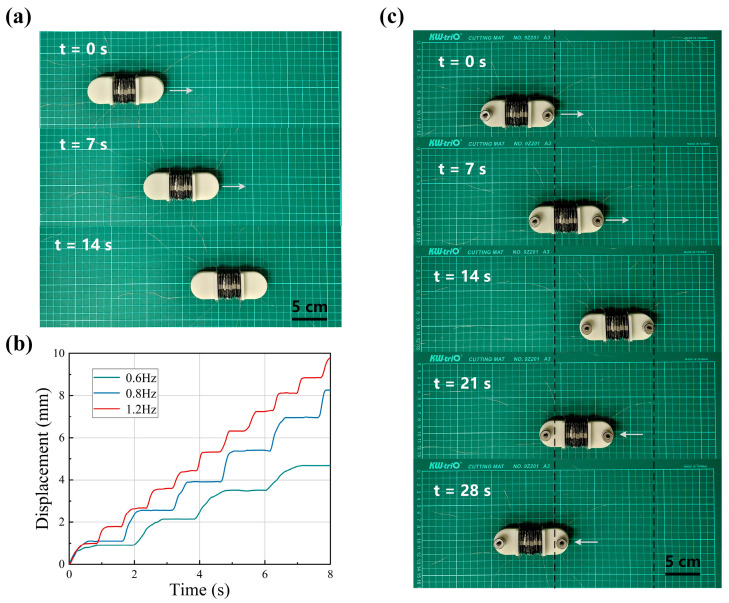
Continuous Motion Experiment of the worm-like robot. (**a**) Crawling locomotion on a PVC flat surface. (**b**) Crawling locomotion on a PVC flat surface. (**c**) Bidirectional locomotion on a moist PVC flat surface with a carried load of 100 g.

Continuous motion experiments were conducted on the robot on a PVC plane based on the control signals described above. The unidirectional motion from t = 0 to 14 s, shown in Figure 9a, demonstrates that the robot maintains stable straight-line propulsion with minimal deviation. The robot’s movement data is obtained by tracking head markers using Tracker video analysis software (version 4.96), which is then used to calculate the robot’s average speed. The trajectory of the robot at different frequencies is shown in Figure 9b. It maintains a stable, continuous motion at all three drive voltage frequencies. Looking at the trajectory, it is clear that the average speed of the robot does not increase linearly with the drive frequency. This behavior is consistent with the expected strain attenuation caused by uneven fluid distribution in the actuator.

In addition to dry surfaces, we also investigated the mobility of the robot on wet surfaces. In preliminary experiments, the bioinspired adhesive surface showed good adhesion in a wet environment. However, under high-frequency voltage driving, the robot’s anchoring module slipped slightly. This was due to insufficient time for the adhesive surface to form a stable wet adhesion liquid film, coupled with the low preload generated by the light weight of the robot’s head and tail modules. A 50 g weight was attached to the shell material of each head and tail module to apply preload and the experiment was repeated. The motion of the robot at a drive frequency of 1.5 Hz is shown in Figure 9c. From t = 0 to 14 s, the robot moves unidirectionally with no significant difference in performance compared to dry surfaces. By adjusting the drive signal, the robot can continuously reverse its motion. After the same time (t = 14–28 s), it returns almost to its initial position.

## 4. Conclusions

This paper presents a worm-like soft robot driven by electrohydraulic actuators. It is capable of steering and bidirectional motion on a plane, as well as rapid propulsion on both dry and wet surfaces. Table 1 presents a detailed comparison of the parameters and performance between our design and conventional worm-like soft robots, emphasizing the robot’s performance and efficiency. Unlike existing actuation methods, the proposed soft robot is inspired by the locomotion mechanism of worms. It incorporates a targeted design for the electrohydraulic actuators and uses a bioinspired adhesive surface with hierarchical polygonal microstructures to actively control adhesion forces, thereby improving the mobility performance of the robot. Thanks to the adhesion-enhancing and friction-reducing design of the anchoring module, our robot requires less mass than other single-segment screw robots. This allows the robot to be both lightweight and capable of carrying a certain load, depending on the application. Experimental data show that the robot exhibits outstanding performance in key metrics such as size design, lightweight construction, and propulsion speed, achieving a maximum speed to body/length ratio of 6.22 min^−1^. This novel soft-bodied crawling robot has promising potential for applications in military reconnaissance, environmental monitoring, and other fields.

In the future, we want to address some of the current limitations of the robot. Currently, the robot’s motion module is limited to movement within a two-dimensional plane. Extending its mobility to three-dimensional space will enable capabilities such as erect movement and obstacle negotiation. In addition, the potential of the robot’s electrohydraulic actuator modules for independent control remains under-utilized. Independent control of different segments could enable multimodal movement, improving the robot’s adaptability to unstructured environments. Moreover, exploiting the soft properties and capacitive self-sensing to achieve autonomous recovery from external disturbances would enhance the robot’s operational capabilities in diverse or unknown environments. In terms of design, additional designs can be implemented based on the current adhesive structure to enhance the adaptability of the adhesive surface in irregular environments, not just on flat surfaces. Modular design and the exploration of multi-segment robots may lead to similar improvements. Further research is also needed in motion modeling and control.

## Figures and Tables

**Table 1:** Comparison with other worm-like soft robots.

Parameter	This Work	Lu et al. [26]	Jung et al. [48]	Mitchell et al. [49]
Actuator type	Electro- hydraulic	DEA	Pneumatic	Electro- hydraulic
Length (mm)	100	150	171	140
Width (mm)	35	40	20	50
Mass (g)	46	8	—	240
Linear velocity (mm s^−1^)	10.36	11.50	12.7	14.75
Velocity/length ratio (min^−1^)	6.22	4.60	4.46	4.60
Velocity/mass ratio (mm (min^−1^ g^−1^))	13.51	86.25	—	3.69
Bidirectional locomotion	Yes	Yes	Yes	Yes
Direction adjustment	Yes	No	No	No
Variable friction	Yes	Yes	Yes	Yes

## Data Availability

Data are contained within the article.
